# Reports of evidence planting by police among a community-based sample of injection drug users in Bangkok, Thailand

**DOI:** 10.1186/1472-698X-9-24

**Published:** 2009-10-07

**Authors:** Nadia Fairbairn, Karyn Kaplan, Kanna Hayashi, Paisan Suwannawong, Calvin Lai, Evan Wood, Thomas Kerr

**Affiliations:** 1 British Columbia Centre for Excellence in HIV/AIDS, St. Paul's Hospital, Vancouver, Canada; 2 Department of Medicine, University of British Columbia, Vancouver, Canada; 3 Thai AIDS Treatment Action Group, Bangkok, Thailand

## Abstract

**Background:**

Drug policy in Thailand has relied heavily on law enforcement-based approaches. Qualitative reports indicate that police in Thailand have resorted to planting drugs on suspected drug users to extort money or provide grounds for arrest. The present study sought to describe the prevalence and factors associated with this form of evidence planting by police among injection drug users (IDU) in Bangkok.

**Methods:**

Multivariate logistic regression was used to identify factors associated with evidence planting of drugs by police among a community-based sample of IDU in Bangkok. We also examined the prevalence and average amount of money paid by IDU to police in order to avoid arrest.

**Results:**

252 IDU were recruited between July and August, 2008, among whom 66 (26.2%) were female and the median age was 36.5 years. In total, 122 (48.4%) participants reported having drugs planted on them by police. In multivariate analyses, this form of evidence planting was positively associated with midazolam use (Adjusted Odds Ratio [AOR] = 2.84; 95% Confidence Interval [CI]: 1.58 - 5.11), recent non-fatal overdose (AOR = 2.56; 95%CI: 1.40 - 4.66), syringe lending (AOR = 2.08; 95%CI: 1.19 - 3.66), and forced drug treatment (AOR = 1.88; 95%CI: 1.05 - 3.36). Among those who reported having drugs planted on them, 59 (48.3%) paid police a bribe in order to avoid arrest.

**Conclusion:**

A high proportion of community-recruited IDU participating in this study reported having drugs planted on them by police. Drug planting was found to be associated with numerous risk factors including syringe sharing and participation in government-run drug treatment programs. Immediate action should be taken to address this form of abuse of power reportedly used by police.

## Background

Illicit injection drug use is associated with significant morbidity and mortality, including infectious disease transmission and overdose [1,2]. Numerous strategies have been implemented to address these harms and deter drug use, including a variety of supply and demand reduction measures [3,4]. Many governments internationally allocate the majority of resources to law enforcement strategies [5-7]. These tactics include arresting individuals who allegedly use drugs or deal drugs in an effort to reduce drug availability and consumption [8,9]. Despite continued investment in these efforts, there is evidence indicating that this type of enforcement often has little impact on the availability and use of drugs [10]. As well, drug law enforcement has been associated with increases in health-related harms among drug users [3,11,12]. For example, policing within drug markets has been associated with HIV risk behaviour among injection drug users (IDU) as a result of reductions in uptake of needle exchange and other harm reduction services [3,11,13-15]. Further, drug law enforcement has been associated with various human rights abuses including illegal searches, unlawful detainment, and assault [16-18].

Thailand, a country with a longstanding HIV epidemic among IDU and prevalence rates as high as 40%, has historically favored strict enforcement as drug policy [19,20]. This approach has led to high rates of incarceration for individuals convicted of possession of illicit substances, and nearly two-thirds of those in prison are drug offenders [21]. Anecdotal reports by drug users in Thailand suggest that abuse of power by police does occur. For example, the planting of drugs on known or suspected drug users for the purposes of extorting money or meeting set quotas for arrest have been noted [22]. In light of these anecdotal reports and the growing concern regarding the adverse consequences of drug law enforcement approaches, we sought to investigate the prevalence and correlates of this form of evidence planting by police among a community-recruited sample of IDU in Bangkok, Thailand.

## Methods

### Participant recruitment

The Mitsampan Community Research Project (MSCRP) is a collaborative research project involving the British Columbia Centre for Excellence in HIV/AIDS (Vancouver, Canada), the Mitsampan Harm Reduction Center (Bangkok, Thailand), the Thai AIDS Treatment Action Group (Bangkok, Thailand), and Chulalongkorn University (Bangkok, Thailand). During the summer of 2008 the research partners designed and undertook a cross-sectional study involving 252 community-recruited IDU. Potential participants were recruited through peer-based outreach efforts and word of mouth. Study participants were invited to attend the Mitsampan Harm Reduction Centre to participate in the study. All participants provided informed consent and completed an interviewer-administered questionnaire eliciting demographic data as well as information about drug use, HIV risk behaviour, experiences with health care, and interactions with police and the criminal justice system. All participants were given a nominal stipend of 250 Thai Baht ($7.50 USD) upon completion of the questionnaire. The study was approved by the Research Ethics Boards of the University of British Columbia and Chulalongkorn University.

### Statistical analyses

The primary aim of this analysis was to document the prevalence and correlates of self-reported evidence planting of drugs by police. Demographic and drug use variables were used to compare IDU who reported ever having drugs planted on them by police with those who did not. These variables of interest were selected on the basis of having some potential explanatory power and included: median age, gender, education level (< secondary school vs. ≥ secondary school), employment status (regular, temporary or self-employed vs. unemployed), participation in illegal income generating activities i.e. drug dealing, theft, or sex trade (yes vs. no), heroin use (yes vs. no), "yaba" (methamphetamine) use (yes vs. no), midazolam use (yes vs. no), use of drugs in combination (yes vs. no), injecting with a used syringe (yes vs. no), lending syringes (yes vs. no), history of non-fatal overdose (yes vs. no), history of incarceration (yes vs. no), history of forced treatment (yes vs. no), and a history of prescription methadone use (yes vs. no). We also asked participants to indicate if they paid the police to avoid arrest (yes vs. no) and, if so, the amount paid in Thai Baht. Multivariate logistic regression was then used to identify those variables independently associated with reporting evidence planting by police. To examine the bivariate associations between each independent variable and the dependent variable of interest, we used the Pearson's Chi-Square test. Fisher's exact test was used when one or more of the cells contained values less than or equal to five. We then applied an *a priori *defined statistical protocol that examined the independent effect of syringe borrowing by fitting a multivariate logistic regression model that included all variables that were significantly associated with the dependent variable at the *p *≤ 0.05 level in univariate analyses. All *p*-values were two-sided.

## Results

In total, 252 IDU were recruited between July and August 2008, including 66 (26.2%) females. The median age was 36.5 years. In total, 122 (48.4%) participants reported a history of having drugs planted on them by police. Table 1 presents the univariate analyses of factors associated with this form of drug planting. As shown here, individuals who reported having drugs planted on them were more likely to report: midazolam use (Odds Ratio [OR] = 3.03; 95% Confidence Interval [CI]: 1.73 - 5.30), combination drug use (OR = 2.20, 95%CI: 1.26 - 3.83), injecting with a used needle (OR = 2.16, 95%CI: 1.27 - 3.65), lending syringes (OR = 2.06, 95%CI: 1.22 - 3.48), non-fatal overdose (OR = 3.17, 95%CI: 1.79 - 5.61), having been in forced drug treatment (OR = 1.99, 95%CI: 1.16 - 3.41), and prescription methadone use (OR = 1.82, 95%CI: 1.10 - 3.02).

**Table 1:** Factors associated with drug planting by police among Thai injection drug users (N = 238)*

Characteristic	Yes n (%) n = 72	No n (%) n = 166	*Odds Ratio* (95% CI)	*p *- value
**Median Age**				
> 36.5 years	63 (52)	63 (48)	0.88 (0.54 - 1.44)	0.614
≤ 36.5 years	59 (48)	67 (52)		
**Gender**				
Female	29 (24)	37 (28)	0.78 (0.45- 1.38)	0.398
Male	93 (76)	93 (72)		
**Education level**				
< Secondary school	45 (37)	58 (45)	1.38 (0.83 - 2.28)	0.213
≥ Secondary school	77 (63)	72 (55)		
**Employment**				
Yes	20 (16)	26 (20)	0.78 (0.41 - 1.49)	0.460
No	102 (84)	104 (80)		
**Illegal Income**				
Yes	8 (7)	6 (5)	1.45 (0.49 - 4.31)	0.503
No	114 (93)	124 (95)		
Heroin Use*				
Yes	115 (94)	119 (92)	1.52 (0.57 - 4.05)	0.404
No	7 (6)	11 (8)		
Yaba Use*				
Yes	82 (67)	79 (61)	1.32 (0.79 - 2.22)	0.288
No	40 (33)	51 (39)		
Midazolam Use*				
Yes	97 (80)	73 (56)	3.03 (1.73 - 5.30)	< 0.001
No	25 (20)	57 (44)		
Combination Drug Use*				
Yes	95 (78)	80 (62)	2.20 (1.26 - 3.83)	0.005
No	27 (22)	50 (38)		
Syringe Borrowing*				
Yes	54 (44)	35 (27)	2.16 (1.27 - 3.65)	0.004
No	68 (56)	95 (73)		
Syringe Lending*				
Yes	55 (45)	37 (28)	2.06 (1.22 - 3.48)	0.007
No	67 (55)	93 (72)		
Non-fatal Overdose*				
Yes	51 (42)	24 (18)	3.17 (1.79 - 5.61)	< 0.001
No	71 (58)	106 (82)		
Incarceration*				
Yes	6 (5)	8 (6)	0.79 (0.27 -2.34)	0.669
No	116 (95)	122 (94)		
Forced Drug Treatment*				
Yes	48 (39)	32 (25)	1.99 (1.16 - 3.41)	0.013
No	74 (61)	98 (75)		
Prescription Methadone Use*				
Yes	63 (52)	48 (37)	1.82 (1.10 - 3.02)	0.019
No	59 (48)	82 (63)		

*Variables refer to ever in the past.

Table 2 presents the multivariate analyses of factors independently associated with evidence planting by police. As shown here, reporting a history of evidence planting was independently and positively associated with midazolam use (Adjusted Odds Ratio [AOR] = 2.84; 95% Confidence Interval [CI]: 1.58 - 5.11), non-fatal overdose (AOR = 2.56; 95%CI: 1.40 - 4.66), syringe lending (AOR = 2.08; 95%CI: 1.19 - 3.66), and having been in forced drug treatment (AOR = 1.88; 95%CI: 1.05 - 3.36).

**Table 2:** Multivariate logistic regression analysis of factors associated with drug planting by police among Thai IDU (N = 238)**

Variable Adjusted	Odds Ratio (AOR)	95% Confidence Interval (95% CI)	*p *- value
Midazolam use*			
(Yes vs. No)	2.84	(1.58 -- 5.11)	< 0.001
History of Overdose*			
(Yes vs. No)	2.56	(1.40 -- 4.66)	< 0.001
Syringe Lending*			
(Yes vs. No)	2.08	(1.19 -- 3.66)	0.010
Forced Drug Treatment*			
(Yes vs. No)	1.88	(1.05 -- 3.36)	0.030

*Variables refer to ever in the past.

**The model was adjusted for syringe borrowing, combination drug use, and prescription methadone use.

Among those who reported an experience of having drugs planted by police, 59 (48.3%) reported paying the police money in an attempt to avoid arrest. The amount paid ranged from 500 - 100,000 Thai Baht (median = 5000 THB; $140 USD).

## Discussion and Conclusion

According to the participants in this study, Thai police commonly plant drugs on IDU, with 50% of participants reporting a history of this form of evidence planting. In multivariate analyses, after extensive covariate adjustment, midazolam injection, non-fatal overdose, syringe lending and participation in forced drug treatment were independently and positively associated with evidence planting by police. In sub-analyses, 48% of IDU reportedly paid police in an effort to avoid arrest following such an occurrence. The amount of money paid varied greatly, with a median amount of 5000 Thai Baht ($140 USD).

Our analysis of self-reported evidence planting helps to corroborate previous anecdotal reports by suggesting that Thai police routinely plant drugs on suspected drug users and dealers [16]. Human rights groups and the United Nations Special Rapporteur on Right to Health have criticized Thailand regarding use of excessive force and brutality as part of its drug enforcement approach, most notably during the "drug war" of 2003 [22]. This particular initiative, purportedly aimed at suppressing drug trafficking and preventing drug use, left an estimated 2,800 people living in Thailand murdered [16,23,24]. During the drug war, the government prepared blacklists of suspected drug users and local officials were required to meet set quotas to reduce the number of people on blacklists, either through arrest or forced drug treatment [16]. Our findings indicate that evidence planting by police is indeed another way in which abuse of power by police may be exerted in Thailand, perhaps for the purpose of maximizing rates of arrests for drug possession or for the simple purposes of extortion.

The association observed in the present study between self-reported drug planting by police and syringe lending is particularly concerning since previous studies have identified policing, especially in the context of "crackdowns", to be a strong predictor of syringe sharing, a behaviour independently associated with HIV infection among Thai IDU [11,25-27]. Though causal relationships can not be inferred in the present study, it may be that after experiencing drug planting, IDU become more fearful of arrest or harm by police and less likely to carry drug-related equipment. Additionally, fear of confrontations with police has previously been identified as leading to a reluctance to visit HIV clinics (where antiretrovirals are distributed) for fear of their drug-using status being reported to police by the clinics, thereby decreasing uptake of services by IDU with HIV [22].

Midazolam, a legal benzodiazepine with potent amnesic and ventilatory depressant effects [28], was found to be independently associated with evidence planting of drugs by police. We postulate that the drowsiness and amnesia associated with benzodiazepine use may allow for easy identification of these IDU by police. Further, it has been argued that IDU may inject in a more hurried and opportunistic fashion due to fear of police, which may be exacerbated in settings where abuse of power by police occurs and lengthy prison sentences are enforced for drug possession. Prospective data is needed to determine if police tactics such evidence planting of drugs may underpin the association between overdose and drug planting observed in the present study [15,29].

Forced drug treatment centers are widespread in Thailand [24,30,31]. Though the temporal relationship in the association between reporting evidence planting by police and having a history of being in forced drug treatment is unclear, we hypothesize that some individuals in drug treatment have had drugs planted on them as police worked to meet set quotas for arrest [22]. Alternately, this association may represent an important breach of confidentiality in that police can identify and target individuals who have previously been in treatment [22].

In conjunction with Thailand's Narcotic Addict Rehabilitation Act, B.E. 2545 (2002) stating that people who are dependent on drugs should be 'treated as patients and not criminals', we recommend Thai drug policy shift focus from one of excessive reliance on enforcement to a health-focused approach, such as through improving access to voluntary and confidential drug treatment centers instead of forced centers [32]. Additionally, urgent action must be taken to reform any policing practices, including tactics potentially used by police such as evidence planting, which violate the human rights of drug users.

Numerous efforts have been made in other settings to change policing practices as a means of reducing the health and social consequences commonly associated with policing that target illicit drug use. Examples include the provision of harm reduction training for police officers, or involving police directly in harm reduction activities [33-35]. While a small number of evaluations have indicated some positive benefits of such efforts, such as increased awareness of health issues and harm reduction among police and greater collaboration among partners, the impacts have generally been modest, and success in achieving many of the more ambitious goals associated with these initiatives has proved difficult [36-38]. Further, the available evidence indicates that substantial barriers to change exist within police structures and cultures [39-41]. As well, while police departments may accept policies that complement public health efforts, the behaviour of individual police officers on the street may deviate from department policies [33,36,39]. Introducing novel methods to address policing practices that compromise health and violate human rights is therefore important. A small number of novel practices have been implemented in the United States and Australia through the use of specialized trainings, public and police surveys, and proactive police oversight mechanisms [42], although there is a clear need for ongoing development in this area.

This study has several limitations. First, self-report was used to gather data, and therefore the results could be susceptible to socially desirable reporting. Though previous research has found self-report by drug users to be sufficiently reliable in descriptions of drug-related problems [43], socially desirable reporting of drug use and risky behaviours as well as memory difficulties remain concerns [44,45]. However, features of this community-based research study, including recruitment and interview administration by peer drug users and paperless consent acquisition, may help to reduce social desirability bias and enhance the reliability of self-report in the present study. Second, the study sample was not randomly selected, and so the findings presented herein may not generalize to other Thai IDU. Finally, our study is cross-sectional in nature and therefore the causal relationships in the observed associations can not be inferred. We recommend further research including longitudinal studies that seek to tease out the temporal relationship between the experience of drug planting and risk behaviours such as overdose and syringe lending. Qualitative research methods could also be used to shed light on the types of circumstances that result in drug planting, as well as the effects of these events on the behaviours of IDU.

In the present study, we observed an alarmingly high rate of reports of evidence planting by police among a community-recruited sample of Thai IDU. Threat from police may contribute to engagement in risk behaviours, such as syringe lending, observed in this study. Immediate action should be taken to address this form of abuse of power and other punitive tactics reportedly used on Thai IDU by police.

## Competing interests

The authors declare that they have no competing interests.

## Authors' contributions

NF participated in the study design, study coordination and drafted the manuscript. KK, KH and PS participated in study design and coordination. CL provided assistance in statistical analysis. EW and TK conceived of the study and participated in its design, coordination, statistical analysis and manuscript preparation. All authors read and approved the final manuscript.

## Pre-publication history

The pre-publication history for this paper can be accessed here:

http://www.biomedcentral.com/1472-698X/9/24/prepub

## References

[B1] Mathers BM Degenhardt L Phillips B Wiessing L Hickman M Strathdee SA Wodak A Panda S Tyndall M Toufik A Global epidemiology of injecting drug use and HIV among people who inject drugs: a systematic review Lancet 2008 372 9651 1733 1745 10.1016/S0140-6736(08)61311-2 18817968

[B2] Shepard CW Finelli L Alter MJ Global epidemiology of hepatitis C virus infection Lancet Infect Dis 2005 5 9 558 567 10.1016/S1473-3099(05)70216-4 16122679

[B3] Friedman SR Cooper HL Tempalski B Keem M Friedman R Flom PL Des Jarlais DC Relationships of deterrence and law enforcement to drug-related harms among drug injectors in US metropolitan areas AIDS 2006 20 1 93 99 10.1097/01.aids.0000196176.65551.a3 16327324

[B4] Maher L Dixon D Policing and public health: Law enforcement and harm minimization in a street-level drug market Brit J Criminol 1999 39 4 488 512 10.1093/bjc/39.4.488

[B5] Moore TJ The size and mix of government spending on illicit drug policy in Australia Drug Alcohol Rev 2008 27 4 404 413 10.1080/09595230802093737 18584391

[B6] De Beck K Wood E Montaner J Kerr T Canada's new federal 'National Anti-Drug Strategy': an informal audit of reported funding allocation Int J Drug Policy 2009 20 2 188 191 10.1016/j.drugpo.2008.04.004 18571396

[B7] Rigter H What drug policies cost: drug policy spending in the Netherlands in 2003 Addiction 2006 101 3 323 329 10.1111/j.1360-0443.2006.01337.x 16499504

[B8] Beyrer C Jittiwutikarn J Teokul W Razak MH Suriyanon V Srirak N Vongchuk T Tovanabutra S Sripaipan T Celentano DD Drug use, increasing incarceration rates, and prison-associated HIV risks in Thailand AIDS Behav 2003 7 2 153 161 10.1023/A:1023946324822 14586200

[B9] Miller CL Firestone M Ramos R Burris S Ramos ME Case P Brouwer KC Fraga MA Strathdee SA Injecting drug users' experiences of policing practices in two Mexican-U.S. border cities: public health perspectives Int J Drug Policy 2008 19 4 324 331 10.1016/j.drugpo.2007.06.002 17997089 PMC2546504

[B10] Wood E Kerr T Small W Jones J Schechter MT Tyndall MW The impact of police presence on access to needle exchange programs JAIDS 2003 34 1 116 118 14501805 10.1097/00126334-200309010-00019

[B11] Cooper H Moore L Gruskin S Krieger N The impact of a police drug crackdown on drug injectors' ability to practice harm reduction: a qualitative study Soc Sci Med 2005 61 3 673 684 10.1016/j.socscimed.2004.12.030 15899325

[B12] Wood E Kerr T Tyndall MW Montaner JS A review of barriers and facilitators of HIV treatment among injection drug users AIDS 2008 22 11 1247 1256 10.1097/QAD.0b013e3282fbd1ed 18580603

[B13] Rhodes T Platt L Sarang A Vlasov A Mikhailova L Monaghan G Street policing, injecting drug use and harm reduction in a Russian city: a qualitative study of police perspectives J Urban Health 2006 83 5 911 925 10.1007/s11524-006-9085-y 16855880 PMC2438598

[B14] Small W Kerr T Charette J Schechter M Spittal P Impacts of intensified police activity on injection drug users: evidence from an ethnographic investigation Int J Drug Policy 2006 17 85 95 10.1016/j.drugpo.2005.12.005

[B15] Wood E Spittal PM Small W Kerr T Li K Hogg RS Tyndall MW Montaner JS Schechter MT Displacement of Canada's largest public illicit drug market in response to a police crackdown CMAJ 2004 170 10 1551 1556 15136548 10.1503/cmaj.1031928 PMC400719

[B16] Not Enough Graves: The War on Drugs, HIV/AIDS, and Violations of Human Rights 2004 16 Human Rights Watch http://www.hrw.org/en/node/12005/section/2 Series 8

[B17] Recalibrating the Regime: The Need for a Human Rights-Based Approach to International Drug Policy 2008 International Harm Reduction Association, Human Rights Watch, Canadian HIV/AIDS Legal Network, Beckley Foundation Drug Policy Programme http://www.idpc.net/php-bin/documents/BFDPP_RP_13_Recal_Regime_EN.pdf

[B18] Do Not Cross: Policing and HIV Risk Faced by People Who Use Drugs 2007 Canadian HIV/AIDS Legal Network http://www.aidslaw.ca/publications/interfaces/downloadFile.php?ref=1080

[B19] Kitayaporn D Uneklabh C Weniger BG Lohsomboon P Kaewkungwal J Morgan WM Uneklabh T HIV-1 incidence determined retrospectively among drug users in Bangkok, Thailand AIDS 1994 8 1443 1450 7818815 10.1097/00002030-199410000-00011

[B20] HIV/AIDS Sentinel Seroprevalence Surveillance Report, 1989-2003 Division of Epidemiology 2004 http://gametlibrary.worldbank.org/FILES/678_HIV%20Seroprevalence%20survey%20Malawi%202003.pdf

[B21] Kalyanasuta K Suriyawong A The Criminal Justice System and Community-Based Treatment of Offenders in Thailand121st International Training Course 2002 61 Tokyo: United Nations Asia and Far East Institute for the Prevention of Crime and Treatment of Offenders 265 293

[B22] Deadly denial: barriers to HIV/AIDS treatment for people who use drugs in Thailand 2007 19 New York: Human Rights Watch 57 http://www.hrw.org/reports/2007/thailand1107/index.htm

[B23] Thailand Narcotics Annual Report 2002 2002 Bangkok: Office of the Prime Minister

[B24] Practical Guideline for Drug Addict Treatment: Regional Sectors 2003 Chapter 1-4 Bangkok: Operational Narcotic Drug Control Center, Ministry of Public Health

[B25] Kitayaporn D Vanichseni S Mastro TD Raktham S Vaniyapongs T Des Jarlais DC Infection with HIV-1 subtypes B and E in injecting drug users screened for enrollment into a prospective cohort in Bangkok, Thailand JAIDS 1998 19 3 289 295 10.1097/00002030-199112000-00014 9803972

[B26] Choopanya K Vanichseni S Des Jarlais DC Plangsringarm K Sonchai W Carballo M Risk factors and HIV seropositivity among injecting drug users in Bangkok AIDS 1991 5 12 1509 1513 10.1097/00002030-200102160-00013 1814333

[B27] Vanichseni S Kitayaporn D Mastro TD Mock PA Raktham S Des Jarlais DC Continued high HIV-1 incidence in a vaccine trial preparatory cohort of injection drug users in Bangkok, Thailand AIDS 2001 15 3 397 405 full_text 11273220 10.1097/00002030-200102160-00013

[B28] Olkkola KT Ahonen J Midazolam and other benzodiazepines Handb Exp Pharmacol 2008 182 335 360 18175099 10.1007/978-3-540-74806-9_16

[B29] Darke S Kaye S Ross J Geographical injecting locations among injecting drug users in Sydney, Australia Addiction 2001 96 241-246 10.1111/j.0887-378X.2004.00304.x 11182868

[B30] Pearshouse R Compulsory Drug Treatment in Thailand: Observations on the Narcotic Addict Rehabilitation Act B.E. 2545 (2002) 2009 Toronto: Canadian HIV/AIDS Legal Network19606547

[B31] Reid G Costigan G Revisiting 'The Hidden Epidemic' 2002 Australia: The Centre for Harm Reduction

[B32] Narcotic Addict Rehabilitation Act BE 2545 2002 http://www1.oncb.go.th/document/narcotics%20addict%20rehabilitation%20act%20B.E.2545%20p91-103.pdf

[B33] Burris S Blankenship KM Donoghoe M Addressing the "Risk Environment" for Injection Drug Users: The Mysterious Case of the Missing Cop Milbank Q 2004 82 1 125 126 15016246 10.1111/j.0887-378X.2004.00304.x PMC2690204

[B34] Forell S Price L Using harm reduction policing within drug law enforcement in the NSW Police Services, Australia Australia 1997 10.1081/JA-120004162

[B35] Grund JP Blanken P Adriaans NF Kaplan CD Barendregt C Meeuwsen M Reaching the unreached: targeting hidden IDU populations with clean needles via known user groups J Psychoactive Drugs 1992 24 1 41 47 10.1016/S0955-3959(02)00031-2 1619521

[B36] Hough M Drug user treatment within a criminal justice context Subst Use Misuse 2002 37 8-10 985 996 10.1016/S0047-2352(00)00069-6 12180574

[B37] Midford R Acres J Lenton S Loxley W Boots K Cops, drugs and the community: establishing consultative harm reduction structures in two Western Australian locations Int J Drug Policy 2002 13 3 181 188 10.1177/1098611103257074

[B38] Smith BW Novak KJ Frank J Travis LF Multijurisdictional drug task forces: an analysis of impacts Journal of Criminal Justice 2000 28 6 543 556 10.1016/S0047-2352(01)00102-7

[B39] Goldstein H Problem-oriented policing 1990 Philadelphia: Temple University Press

[B40] Paoline EA Shedding light on police culture: An examination of officers' occupational attitudes Police Quarterly 2004 72 2 205 236

[B41] Zhao J Lovrich NP Robinson TH Community policing - Is it changing the basic functions of policing? Findings from a longitudinal study of 200+ municipal police agencies J Crim Just 2001 29 365 377

[B42] Prenzler T Ronken C A survey of innovations in the development and maintenance of ethical standards by Australian police departments Police Practice & Research 2003 4 2 149 161

[B43] Darke S Self-report among injecting drug users: a review Drug Alcohol Depend 1998 51 3 253 263 9787998 10.1016/s0376-8716(98)00028-3

[B44] Johnson T Fendrich M Modeling sources of self-report bias in a survey of drug use epidemiology Ann Epidemiol 2005 15 5 381 389 15840552 10.1016/j.annepidem.2004.09.004

[B45] Latkin CA Vlahov D Socially desirable response tendency as a correlate of accuracy of self-reported HIV serostatus for HIV seropositive injection drug users Addiction 1998 93 8 1191 1197 9813900 10.1046/j.1360-0443.1998.93811917.x

